# Transfer of Visual Learning Between a Virtual and a Real Environment in Honey Bees: The Role of Active Vision

**DOI:** 10.3389/fnbeh.2018.00139

**Published:** 2018-07-13

**Authors:** Alexis Buatois, Clara Flumian, Patrick Schultheiss, Aurore Avarguès-Weber, Martin Giurfa

**Affiliations:** Research Centre on Animal Cognition, Center for Integrative Biology, CNRS, University of Toulouse, Toulouse, France

**Keywords:** visual conditioning, transfer of learning, virtual reality, Y-maze, honey bees, insect cognition

## Abstract

To study visual learning in honey bees, we developed a virtual reality (VR) system in which the movements of a tethered bee walking stationary on a spherical treadmill update the visual panorama presented in front of it (closed-loop conditions), thus creating an experience of immersion within a virtual environment. In parallel, we developed a small Y-maze with interchangeable end-boxes, which allowed replacing repeatedly a freely walking bee into the starting point of the maze for repeated decision recording. Using conditioning and transfer experiments between the VR setup and the Y-maze, we studied the extent to which movement freedom and active vision are crucial for learning a simple color discrimination. Approximately 57% of the bees learned the visual discrimination in both conditions. Transfer from VR to the maze improved significantly the bees’ performances: 75% of bees having chosen the CS+ continued doing so and 100% of bees having chosen the CS− reverted their choice in favor of the CS+. In contrast, no improvement was seen for these two groups of bees during the reciprocal transfer from the Y-maze to VR. In this case, bees exhibited inconsistent choices in the VR setup. The asymmetric transfer between contexts indicates that the information learned in each environment may be different despite the similar learning success. Moreover, it shows that reducing the possibility of active vision and movement freedom in the passage from the maze to the VR impairs the expression of visual learning while increasing them in the reciprocal transfer improves it. Our results underline the active nature of visual processing in bees and allow discussing the developments required for immersive VR experiences in insects.

## Introduction

The visual capacities of honey bees have been intensively investigated for more than a century. Since the pioneer experiments by Karl von Frisch (von Frisch, 1914) and Mathilde Hertz (e.g., Hertz, 1935) on honey bee color and pattern vision, respectively, many scientists have used simple behavioral protocols to access different aspects of bee vision. These protocols rely on the fact that free-flying bees learn rapidly to choose and land on visual targets that have been associated with a reward of sucrose solution (Giurfa and Menzel, 1997; Srinivasan and Zhang, 2004; Srinivasan, 2010; Avarguès-Weber et al., 2011).

In the last decades, many experiments on bee visual perception and learning have been performed in Y-mazes as this type of setup allows a proper control of the distance at which a decision based on visual information is made. It is thus possible to determine the visual cues accessible to the bees upon decision (van Hateren et al., 1990; Giurfa et al., 1996). Free-flying bees can be easily trained to enter such mazes to collect sucrose solution upon appropriate choice of trained colors and patterns in simple or complex learning sets (e.g., Giurfa et al., 1996, 2001; Avarguès-Weber et al., 2010, 2011, 2012). These experiments are also possible in the case of walking bees that are presented with visual discriminations within mazes of small size in which flight is precluded, thus adding further possibilities for behavioral control (e.g., Chittka, 1998; Zhang et al., 1998; Buatois et al., 2017). Using such controlled conditions, researchers showed that bees do not only learn simple discriminations between colors and/or shapes associated with different reinforcements but also learn higher-order discriminations in conceptual and categorization problems (see reviews in Srinivasan and Zhang, 2004; Benard et al., 2006; Srinivasan, 2010; Avarguès-Weber et al., 2011; Dyer, 2012; Giurfa, 2013).

The neural underpinnings of these capacities, both for simple and higher-order learning, remain, however, elusive. On the one hand, the use of free-flying or walking bees precludes the use of invasive methods to obtain more in-depth information about the neural mechanisms involved in visual learning. On the other hand, attempts to train harnessed bees to associate visual stimuli with sucrose reward have been mostly unsatisfactory, at least when using the proboscis extension response (PER) as the behavioral readout of visual learning and memory formation. While harnessed bees easily learn odor-sucrose associations and extend their proboscis to odors previously rewarded (Takeda, 1961; Bitterman et al., 1983; Giurfa and Sandoz, 2012), their learning of colors in the same conditions is usually poor, even for simple color discrimination tasks (Hori et al., 2006; Mota et al., 2011; Dobrin and Fahrbach, 2012; Balamurali et al., 2015; Avarguès-Weber and Mota, 2016). Similarly, when movements of striped patterns are associated with sucrose reward in order to condition PER to a forward or backward movement, learning is typically slow and deficient (Hori et al., 2007). Attempts to condition antennal movements to visual stimuli rather than PER were also disappointing: while bees exhibit stereotyped and specific antennal movements to the ventro-dorsal movement of a striped pattern (Erber et al., 1993), enhancing these responses via pairing with sucrose yielded only partial success: improvement occurred only for certain directions of stripe-pattern movement and in no case bees could learn to discriminate between opposite directions (Erber and Schildberger, 1980).

A potential explanation for the deficit resulting from preparations in which bees are fully immobilized, as required by PER conditioning protocols, is the absence or limitation of active vision, which might be essential to learn visual targets. In active vision, an observer varies its viewpoint to investigate the environment and extract more information from it. This strategy is used for example by flying bees and wasps (Zeil, 1993a,b, 1997; Zeil et al., 1996; Srinivasan and Zhang, 2004) to extract the borders of objects for better recognition (Lehrer and Srinivasan, 1993; Hempel de Ibarra and Giurfa, 2003) via a series of flight maneuvers. Addressing this hypothesis requires manipulating the possibilities of active vision, i.e., the freedom of movement of a bee solving visual discriminations.

Studying the visual performances of tethered insects offers the possibility of controlling both their visual environment and their freedom of movement. For instance, in the so-called “flight simulator” an insect glued to a small hook of copper wire and attached to a torque meter flies stationary in the middle of a cylindrical arena displaying different visual patterns. In this device, originally conceived for fruit flies (Götz, 1964; Heisenberg and Wolf, 1988; Wolf and Heisenberg, 1991), the rotational speed of the arena is proportional to the fly’s yaw torque around its vertical body axis under closed loop conditions. This allows the fly to stabilize the rotational movements of the panorama and to establish flight directions with respect to visual patterns displayed on the cylinder. The flight simulator allowed to study visual landmark learning in several neurogenetic *Drosophila* mutants, thus uncovering the neural and molecular bases of some forms of visual learning and memory (Liu et al., 1999, 2006; Brembs and Heisenberg, 2000; Tang and Guo, 2001; Xi et al., 2008; Pan et al., 2009).

The study of visual learning and memory in a flight simulator has not been possible until now in the case of honey bees. The closest attempt consisted of an analysis of the body posture of a tethered bee flying stationary in the middle of a visual arena made up of four LCD monitors disposed in a diamond-like arrangement and displaying a moving panorama (Luu et al., 2011). The monitors provided a simulation of image variation as the insect flies. It was shown that the bee raised its abdomen progressively higher as the simulated speed of the image increased and tilted it down when the visual motion stimulus stopped. This behavior termed “streamlining response” is a spontaneous response to motion cues “*en route*” to the goal. It does not involve the learning of visual cues and occurs in a context different from the close-up recognition of visual targets learned in association with food reward, when the animal is about to land.

A better solution for the study of learning of visual targets in tethered bees is provided by the use of treadmills onto which bees walk stationary while being exposed to visual targets paired with food reward or with punishment (Buatois et al., 2017; Rusch et al., 2017; Schultheiss et al., 2017). In this kind of setup, closed loop conditions allowed creating a virtual environment in which the bee’s responses are tracked and used to update the virtual environment in real time, thus creating an experience of immersion within this virtual reality (VR; Buatois et al., 2017; Rusch et al., 2017; Schultheiss et al., 2017).

Comparison of performances between this kind of device and Y-mazes offers the possibility of addressing the role of active vision in visual learning. In a Y-maze, full freedom is granted during visual learning while in the treadmill, movements are constrained by tethering the bee to avoid its escape from the setup. Although a tethered bee may walk in any intended direction, as a bee walking in a Y-maze, the physical presence of the tether creates a higher resistance against movements. Thus, additional forces are needed for the animal to achieve a displacement towards a goal (Catton et al., 2007). Moreover, slight asymmetries in the positioning of the tether with respect to the longitudinal axis of the body may favor movements on the side opposite to the tether (Catton et al., 2007), thus affecting the possibility of symmetrical active vision.

Here, we performed a comparative analysis of visual learning in honey bees placed in these two experimental conditions. We used a small Y-maze, where freely walking bees experienced visual stimuli projected onto its back walls, and a VR setup, where tethered bees walking stationary on a treadmill experienced the same visual stimuli projected onto a semi-circular screen placed in front of them. In the latter case, the bee movements constantly updated the visual panorama accordingly (closed-loop conditions). We conditioned independent groups of bees in parallel, either in the VR setup or in the maze, and compared their learning of a color discrimination. After training, each group was transferred to the alternative condition to determine whether VR and maze learning are robust to a change in context. In doing this, we analyzed if restricting movement freedom (from the maze to VR) or enhancing it (from VR to the maze) affected transfer performances and thus discrimination success. Our results show that bees mastered equally well the visual discrimination in both the Y-maze and the VR setup despite obvious differences in movement freedom and in the possibility of performing active vision. Transfer between both contexts affected the expression of learning in an asymmetric way: granting the bees with a greater opportunity for active vision improved visual performances while diminishing it impaired them. We discuss the learning strategies employed by the bees in both contexts and how to achieve better immersive VR experiences in the case of insects.

## Materials and Methods

### Animals

**Figure 1:**
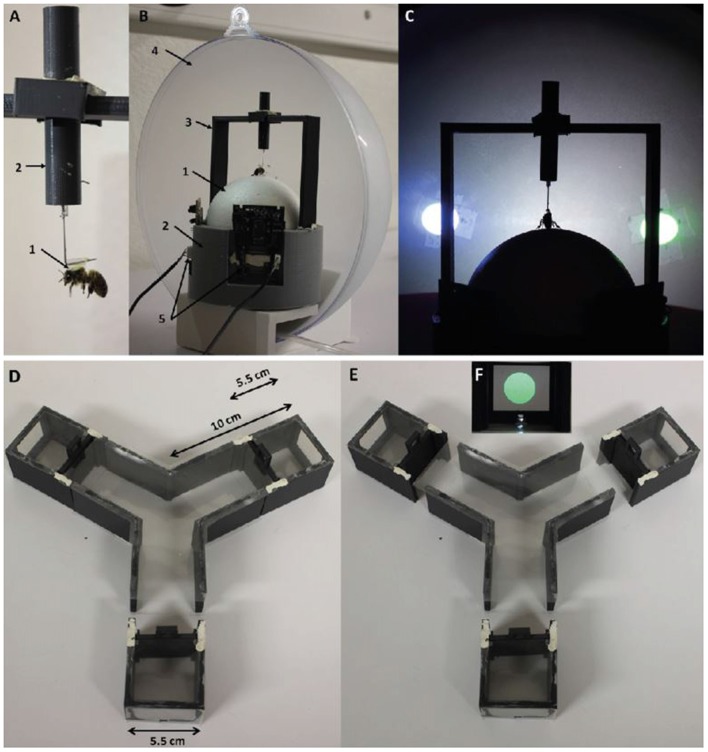
Experimental setups. The virtual reality (VR) setup **(A–C)** and the Y-maze **(D–F)**. **(A)** A bee tethered by the thorax by means of a vertical attachment (1) made of a custom-built tag (1) and an L-shaped metal piece glued to the thorax. The metal piece was enclosed in a plastic cylinder (2), which allowed its vertical displacement and thus the accommodation of the bee on the surface of the treadmill (2). **(B)** Global view of the VR system. The polystyrene ball (1) floated on a constant airflow provided at the basis of a ball support (2). The tethered bee was placed on the ball thanks to a holding support (3). The apparatus is placed behind a semi-spherical opaque screen (4) on which visual stimuli were projected. Two optic-mouse sensors (5) were placed on the ball support, at 90° of each other to record the ball movements. The setup translates the movements of the walking bee into rotations of the ball. **(C)** Front view of the setup during a conditioning trial. The tethered bee walking stationary faced the two-colored discs presented at −45° and +45° of its longitudinal axis. **(D)** Top view of the Y-maze. Each arm was connected to a removable box with a sliding door, which allowed displacing an enclosed bee from an arm to another. Arms were 10 cm in length, 4 cm in height and 5.5 cm in width. Each box had a length of 5.5 cm. **(E)** Top view of the maze showing the disconnected boxes and how they could be interchanged between arms of the maze. **(F)** Front view of the inside of a box. A color disc was projected by the video projector onto the article screen placed at the end of each box.

Honey bees (*Apis mellifera*) were obtained from the apiary located at the campus of the University of Toulouse. The experiments were performed with honey bees as animal subjects. No legal requirements exist in the case of insect experiments. We have nevertheless employed procedures ensuring cautious handling of the experimental subjects and minimizing the number of individuals per experiment. Only non-fed foragers caught upon landing on a gravity feeder filled with a 0.9 M sucrose solution were used in our experiments to ensure high appetitive motivation. Once caught, each bee was anesthetized by cooling it on ice for 3 min. The thorax was then shaved to improve the fixation of a custom-built tag with UV-cured dentine (Figure 1A), which allows to tether the bee during the VR experiment. Bees were fed with 4 μl of 0.9 M sucrose solution and kept for 3 h in the laboratory before starting the experiments in order to homogenize their appetitive motivation. Feeding was achieved by means of a toothpick in the case of bees assigned to VR training, while it was done using an Eppendorf in the case of bees assigned to Y-maze training. During the 3 h period, bees assigned to VR training were placed individually on miniature treadmills while bees assigned to Y-maze training were placed individually in the starting box of the maze (see below) to allow familiarization with their respective setup.

### Virtual Reality Apparatus

The apparatus (Figures 1B,C) is composed of a spherical treadmill on which a tethered bee walked stationary, and a video projection system displaying visual stimuli in front of the bee. The treadmill consists of a polystyrene ball (diameter: 10 cm, weight: 8 g, Figure 1B) positioned on a 3D-printed support (Figure 1B) and floating on a constant air flow produced by an air pump (air flow: 555 ml/s; Aqua Oxy CWS 2000, Oase, Wasquehal, France). The treadmill was placed in front of a semi-spherical semi-transparent plastic screen (diameter: 29 cm, distance to the bee: 10 cm, Ballkit, Varennes, France, Figure 1B) coated with matt picture varnish (Pébéo, Gemenos, Italy). Visual stimuli were projected onto the screen from behind using a video projector (Acer k135i, Roissy, France).

All VR experiments were done under closed-loop conditions, i.e., rotations of the ball generated by the walking activity of the tethered bee displaced the visual stimuli accordingly on the screen. To this end, the movements of the ball were recorded by two infrared optic-mouse sensors (Logitech M500, 1000 dpi, Logitech, Lausanne, Switzerland, Figure 1B), which were placed on the ball support, at 90° from each other. The rotational speed of the ball around the vertical axis was calculated with a LabVIEW (National Instruments, Austin, TX, USA) custom software to account for the directional walking movements of the bee (“instant heading”; one data point every 250 ms) using the following equation.

$$Instant\;heading=-\left(\frac{\frac{X1+X2}{2}}{5700}\right)*\left(\frac{25.4*180}{R\pi }\right)$$

X1 and X2 are, respectively, the translational movement in dots recorded in the horizontal axis of each sensor and 5700 is the sampling rate of the sensors in dots/inch. Multiplying the obtained value by 25.4 allows conversion into millimeters while dividing it by 2πR (with R being the radius of the ball) converts the measured distance from millimeters into radians. Finally, multiplying by 180/π converts radians to degrees.

These values were used by the software to rotate the angular position of the stimuli on the screen proportionally to the movement of the bee (0° being the initial position, −180° the left extremity and 180° the right extremity). In order to decrease the speed of image movement and achieve a proper gain control, the software was configured in such a way that 2° of ball rotation correspond to 1° of stimulus rotation. Thus, in our graphic representations of the bees’ turning activity, a vector pointing towards +90° (circular plot) represents a bee oriented towards a visual stimulus located at 45° to the right of the central axis of the bee body.

### Y-Maze Apparatus

The maze (Figures 1D,E) consisted of three PVC arms defining a Y. Each arm had a length of 10 cm, a height of 4 cm and a width of 5.5 cm. At the end of each arm, detachable boxes with the same section (4 × 5.5 cm) allowed replacing the bee at the starting position of the maze after each choice, thus facilitating further data collection and a better control of the experimental time parameters. The same visual cues used in the VR setup were projected onto the back walls of the detachable boxes (Figure 1F). The back walls were made of transparent article. Both the roof and the floor of the maze were made of thin transparent plastic to allow the passage of light. The setup was placed on an infrared light-emitting platform. Experiments were recorded with an infrared camera (acA1300-60 gm, 60 fps, 1.3 MP, Basler, Ahrensburg, Germany) equipped with light filters to remove the light wavelengths of the stimuli in the videorecordings and facilitate the tracking of the bee. Video recordings were afterwards analyzed using the EthoVision 12 software (Noldus, Wageningen, Netherlands).

### Visual Stimuli

The visual stimuli that bees had to discriminate in both setups were a blue disc (RGB: 0, 0, 255, with dominant wavelength at 450 nm) and a green disc (RGB: 0, 51, 0, with dominant wavelength at 530 nm) displayed on a black background (RGB: 0, 0, 0). Their spectral curves, and their chromatic and achromatic properties are shown in Supplementary Figure S1 and Supplementary Table S1, respectively. Their intensity, measured at the level of the bee eye, was 3363 μWatt/cm^2^ and 2950 μWatt/cm^2^, respectively. These values were chosen to suppress an original attraction of the bees towards the green light detected in preliminary assays. The two discs were 2 cm in diameter. They displayed the same total area (3.14 cm^2^) and subtended the same visual angle to the bee eyes (11.42°) in both setups (VR and Y-maze).

### Reinforcements

The positive reinforcement was a 0.9 M sucrose solution while the negative reinforcement was a 60 mM quinine solution (Buatois et al., 2017). During VR experiments, reinforcements were delivered by means of a toothpick to the antennae and then to the proboscis (see below for more details). In the Y-maze, reinforcements were provided in small Eppendorf tube covers (Eppendorf tube 3810x, Hamburg, Germany) located at the end of the arms, in association with the projected color discs. They contained 4 μl of solution, a volume that was chosen to correspond to the amount delivered by the toothpick during the VR experiments according to a preliminary quantification.

### Training and Testing Protocol

#### Experiment 1: From VR to the Y-Maze

**Figure 2:**
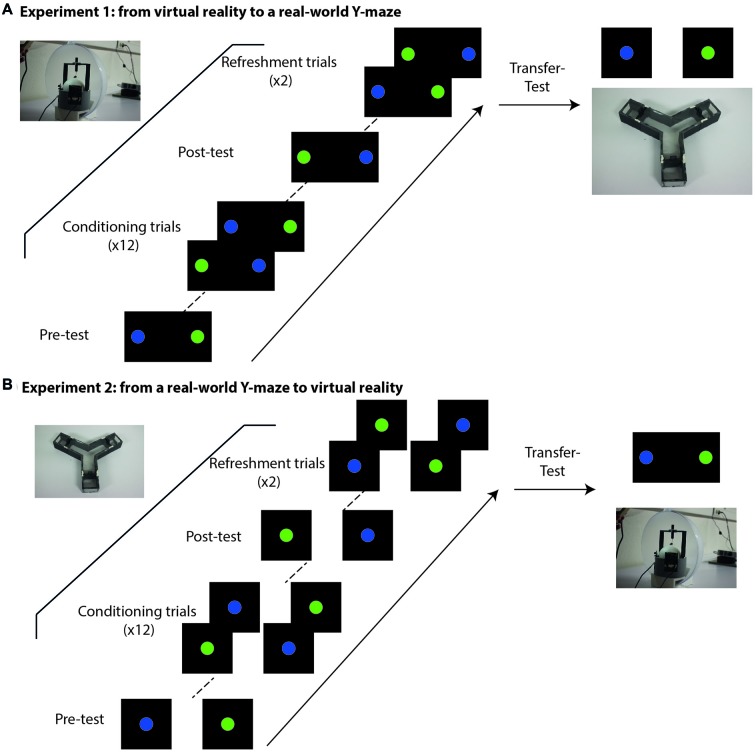
Experimental schedule at the VR setup **(A)** and at the Y-maze **(B)**. **(A)** Experimental sequence of Experiment 1. Bees started with a pre-test in which both colored discs were shown in the absence of reinforcement to check for spontaneous preferences and determine the CS+ (the color less preferred) and the CS− (the color more preferred) for the conditioning procedure. The pre-test was followed by 12 conditioning trials in which both colored discs were shown simultaneously and associated with sucrose solution (CS+) or quinine solution (CS−). After conditioning, a post-test in which both stimuli were shown simultaneously without reinforcement allowed to determine whether bees learned the visual discrimination. Two refreshment trials with reinforcement were performed after the post-test and before the transfer to the Y-maze to avoid extinction of the learned information. Pre-test, conditioning and post-test were performed in closed-loop conditions, i.e., the movements of the bee controlled the visual cues displayed on the screen in front of it. After the post-test, bees were transferred to the Y-maze in which both stimuli were presented in different arms of the maze. Bees were tested for transfer of discrimination learning to this new context. **(B)** Experimental sequence of Experiment 2. The schedule was similar to that of Experiment 1 with the difference that pre-test, training and post-test took place in the Y-maze where the bee movements were not constrained. Colored discs were presented on the article walls at the end of the maze arms. After the post-test, bees were transferred to the VR setup where they could see both colored discs simultaneously.

Conditioning was performed in the VR setup (Figure 2A). Bees were then transferred to the Y-maze to determine whether the change in context, with its associated increase in movement freedom and possibility of active vision, changed the bees’ performance.

The experiment started in the VR setup with a “pre-test” performed to determine the spontaneous stimulus preference of each bee. During this pre-test, the stimuli to be discriminated were presented simultaneously at 45° on each side of the bee’s body axis for 30 s and without reinforcement. The position (left or right) of the blue and green discs was randomized from bee to bee. During the 30 s, the position of the stimuli was constantly updated by the movements of the bee on the treadmill. In order to define which stimulus would act as CS+ and as CS− in the subsequent training, we recorded the time spent by each bee fixating either the blue or the green disc. The stimulus that was fixated longer became the CS− and was reinforced negatively with quinine solution. The stimulus that was less fixated became the CS+ and was reinforced positively with sucrose solution. If the bee did not make any choice during the pre-test, CS+ and CS− were assigned randomly. Besides the fixation time, we also recorded the first choice made by each bee when facing both stimuli (see below, “Statistical Analysis” section). When the pre-test was concluded, a black screen was displayed for 1 min before starting the conditioning protocol.

Each tethered bee was trained in closed-loop conditions to discriminate the blue from the green disc based on their different reinforcement outcome (differential conditioning). Training consisted of a succession of 12 trials, each 30 s in duration, separated by an intertrial interval of 1 min. During each trial, the bee was presented simultaneously with both stimuli, the CS+ paired with 0.9 M sucrose solution and the CS− with 60 mM quinine solution. Stimuli were displayed during at most 30 s and appeared at the start of the trial at −45° (left) and +45° (right) from the bee’s body axis. The stimulus side of CS+ and CS− was varied from trial to trial and the side sequence was the same from bee to bee (G_R_/G_L_/G_L_/G_R_/G_L_/G_R/_G_R_/G_L_/G_R_/G_L/_G_L_/G_R_; with G: green, R: right and L: left; G_R_ means green disc displayed on the right, i.e., blue disc displayed simultaneously on the left). When the bee oriented towards a CS and centered it on the screen due to the closed loop conditions (0° from the bee’s body axis), the CS remained stationary at this position during 8 s to facilitate reinforcement delivery and its association with a plain frontal view of the CS. Sucrose solution was then provided on the antennae using a toothpick. This stimulation triggered proboscis extension, which allowed us to feed the bee. Quinine was provided directly to the proboscis. After the end of the 8-s period, the stimulus was turned off and was replaced by the black background, which was displayed to the bee during intertrial intervals. The bees never moved while the CS was stationary in front of them or while being reinforced. This procedure ensured that all bees experienced the same reinforcement duration.

One minute after the end of the last conditioning trial, the trained bee was subjected to a “post-test” during which it was again presented during 30 s with the CS+ and the CS− simultaneously, but in the absence of reinforcement. As in the pre-test, during the 30 s, the position of the stimuli was constantly updated by the movements of the bee on the treadmill. We recorded the first choice of each bee and the time spent fixating each CS. This post-test allowed verifying if the bee’s original stimulus preference recorded during the pre-test was modified because of learning.

Once the post-test was finished, each bee was subjected to two refreshment trials to avoid extinction and then to a transfer test in the Y-maze. To this end, the bee was taken away from the VR setup, and after removing its tether, it was placed in the departure box of the Y-maze for 1 min. The bee was now free to move and the transfer-test started when both stimuli, CS+ and CS−, were displayed simultaneously, each one in one arm of the maze. The bee was free to move during 30 s during which it could choose the stimuli presented without reinforcement. The left/right position of the stimuli was varied within the maze. The choice behavior of the bee was then recorded.

#### Experiment 2: From the Y-Maze to VR

In the Y-maze, the bees underwent the same conditioning protocol as in the VR setup, i.e., preference testing in a pre-test, subsequent CS assignment, conditioning during 12 trials and learning assessment in a post-test were performed following the same schedules and timing (Figure 2B). Both visual stimuli were projected simultaneously onto the article walls at the end of the maze arms. The main difference with the previous experiment is that bees were free to walk within the maze during trials.

The experiment started with a “pre-test” performed to determine the spontaneous stimulus preference of each bee. During this pre-test, the bee could freely walk between the arms displaying the blue and the green discs without reinforcement during 30 s. The position (left or right) of the blue and green discs was randomized from bee to bee. We recorded the time spent by each bee within each arm. The stimulus that was more attractive to the bee (i.e., more time spent in its associated arm) became the CS− and was reinforced negatively with quinine solution during the subsequent training. The stimulus that was less attractive (i.e., less time spent in its associated arm) became the CS+ and was reinforced positively with sucrose solution during the training. If the bee did not make any choice during the pre-test, CS+ and CS− were assigned randomly. Besides the fixation time, we also recorded the first choice made by each bee when facing both stimuli (see below, “Statistical Analysis” section). When the pre-test was concluded, the conditioning protocol started.

During training, the bee was subjected to 12 trials during which both the CS+ and the CS− were made available. In each trial, the bee left the starting arm to enter one of the boxes displaying either the CS+ or the CS−. As the bee was familiar with the Eppendorf containing the sucrose solution, which was used to feed it at the beginning of the experiment (see above, “Animals” section), it found the reinforcement rapidly. When the bee touched the Eppendorf cover, a sliding door trapped it in the compartment. The bee was left in this detachable compartment during 8 s, a period that was enough to consume the 4 μl of sucrose solution associated with the rewarded color. Between trials, the bee was kept in the dark within the detachable box in which it was trapped. The box was then translocated to the starting point of the maze where the bee could be released to reinitiate a new stimulus choice.

After completing the training, a post-test was performed in which the first choice and the time spent within each CS arm was recorded during 30 s (see below, “Statistical Analysis” section). At the end of the post-test, the bee was subjected to two refreshment trials in the maze to avoid extinction. Afterwards, it was captured, attached to the tether and placed in the VR setup for the transfer-test. The bee was then presented with the CS+ and the CS−, which were displayed at ± 45° of its body axis in the absence of reinforcement and in open-loop conditions. Presentation lasted 30 s and the choice behavior of the bee was then recorded.

### Statistical Analysis

#### Experiment 1: From VR to the Y-Maze

During the pre-test, the first choice of the bee (i.e., the first image centered on the screen, aligned with the axis of the bee’s body) was categorized as “choice of the green disc” or “choice of the blue disc.” If the bee did not fixate a CS, its performance was categorized as “no-choice.” During the conditioning trials, post-test and transfer test, the first choice of the bee was categorized as “choice of the CS+” (i.e., choice of the sucrose-reinforced stimulus) or “choice of the CS−” (i.e., choice of the quinine-reinforced stimulus). If the bee did not fixate either stimulus, its performance was recorded as a “no-choice”. In the transfer test, the first choice, which occurred in the Y-maze, corresponded to the first arm displaying a visual stimulus visited by a bee.

Individual data were converted into a binomial format (0 or 1) to calculate the proportions of bees that chose the CS+, the CS− or made no choice. Each bee was assigned to a unique category. For instance, a bee choosing the CS+ was quantified as (1, 0, 0) for choice of the CS+, choice of the CS− and no-choice, respectively. Data were bootstrapped to plot these proportions ± their corresponding 95% confidence interval. Proportions were compared by means of a generalized linear mixed model (GLMM) for binomial family in which the individual identity (Bee) was considered as a random factor (individual effect). The Tukey method was used for multiple comparisons; z values with corresponding degrees of freedom are reported throughout for this kind of analysis.

The angular positions of the stimulus on the screen were recorded during the pre- and post-test, and their distribution was analyzed using circular statistics. To test whether angular positions were uniformly distributed, we used a Rayleigh Test of Uniformity for General Unimodal Alternative (Rayleigh test), which assesses the significance of the mean resultant length. A Rayleigh Test of Uniformity for Specified Mean Direction (V-test) was used to assess the departure of our data from the specific directions defined by the angular position of the stimuli (±45° on the screen, which translated into ideal angular orientations of ±90°; see above, “Virtual Reality Apparatus” section). Finally, to compare the angular means obtained in the pre- and the post-test, a Watson-Wheeler test was performed.

We also quantified the time spent fixating the stimuli during the pre- and the post-test. Mean values were compared to a theoretical fixation time of 0 s using a one-sample Mann Whitney test. The fixation times of CS+ and CS− were compared using a Wilcoxon *U* rank test.

#### Experiment 2: From the Y-Maze to VR

First-choice categorization was similar as in Experiment 1: during the pre-test, the first choice of an arm displaying a visual stimulus was categorized as “choice of the CS1” or “choice of the CS2.” The absence of choice was recorded as “no-choice.” During the conditioning trials, post-test and transfer test, the first choice of the bee was categorized as “choice of the CS+” (i.e., choice of the sucrose-reinforced stimulus) or “choice of the CS−” (i.e., choice of the quinine-reinforced stimulus). If the bee did not make any choice, its performance was categorized as “no-choice.” In the transfer test, the first choice, which occurred in the VR setup, corresponded to the first CS fixated by a bee.

Individual data were converted into a binomial format (0 or 1) to calculate the proportions of bees, which chose the CS+, the CS− or made no choice (see above). Data were treated and analyzed as described for Experiment 1.

Choice performance in the Y-maze could be further described using a heat map, which represents the normalized mean time spent in a given region of the maze during the pre-test and the post-test. Heat maps were obtained using the EthoVision XT tracking system (Noldus, Wageningen, Netherlands). Moreover, the times spent in the arms displaying the CS+ and the CS− during the pre- and post-test were compared against a theoretical time of 0 s by means of a one-sample Mann Whitney test. The times spent within each arm were compared by means of a Wilcoxon *U* rank test.

#### Experiments 1 and 2: Transfer-Test Performances

To evaluate transfer-test performances, we focused exclusively on learners and non-learners, based on post-test performances (i.e., bees that chose the CS+ and bees that chose the CS−, respectively). Bees that did not make a choice were not included in this analysis. We determined whether the proportions of learners and non-learners changed in the transfer-test with respect of those obtained in the post-test. To evaluate the significance of change, we used a McNemar test.

All statistical analyses were done using the R 3.2.3 software (R Core Team 2016^1^). Packages lme4 and lsmeans were used for GLMMs, with Tukey’s method for multiple comparisons. Circular and CircStats were used for circular graphics and circular statistics.

## Results

### Experiment 1: From VR to the Y-Maze

#### Performance in VR

**Figure 3:**
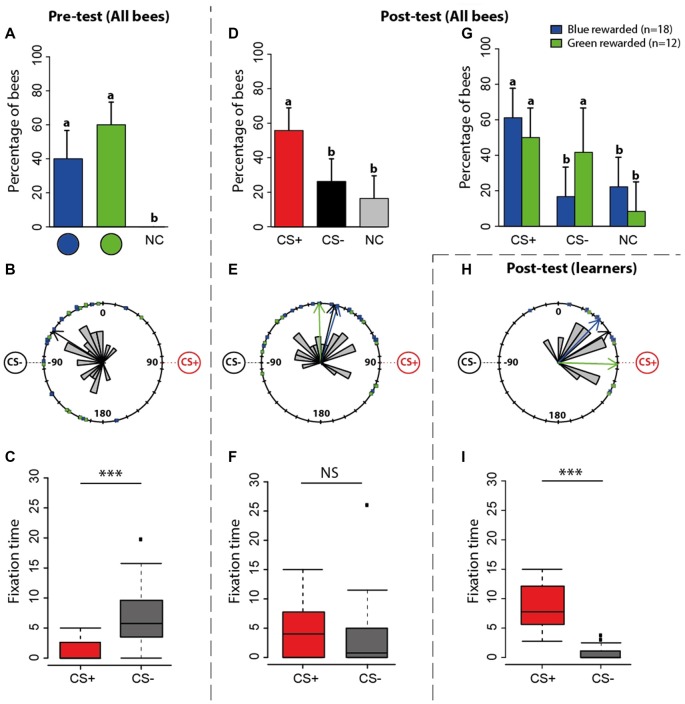
Experiment 1: spontaneous preferences (pre-test) and learning-induced preferences (post-test) at the VR setup. **(A)** Percentage of bees (*n* = 30) choosing first either the green disc, the blue disc or not making a choice (NC) during the pre-test. The 95% confidence interval is shown in each case. **(B)** Circular distribution of orientation vectors of bees during the pre-test. Note that while the stimuli appeared at 45° to the left and right of the main axis of the bee body, they appear at 90° to the left and right in these circular plots because 2° of ball rotation corresponded to 1° of stimulus rotation. Thus, a vector pointing towards 90° represents a bee oriented towards a visual stimulus located at 45° to the right of the central axis of the bee body. The black arrow inside the distribution is the mean resultant vector of the group of bees (*n* = 30, *n* < 0.0001). The blue arrow shows the mean resultant vector of bees preferring the blue disc (*n* = 12, *p* < 0.0001); the green arrow shows the same for bees preferring the green disc (*n* = 18, *p* < 0.0001; here blue and green arrows fully coincide with the black arrow and cannot be seen). Although at this stage of the experiment there is neither a CS+ nor a CS−, we use these terms to indicate the stimuli that will become CS+ and CS− during the subsequent training. **(C)** Fixation time (in seconds; median, quartiles and outliers) of the CS+ (red boxplot) and of the CS− (gray boxplot) during the pre-test. The terms CS+ and CS− are used here in the sense indicated above. **(D)** Percentage of bees (*n* = 30) choosing first either the CS+, the CS− disc or not making a choice (NC) during the post-test. The 95% confidence interval is shown in each case. **(E)** Circular distribution of orientation vectors of bees during the post-test. The black arrow inside the distribution is the mean resultant vector of the entire group of bees (*n* = 30, *p* = 0.18). The blue arrow shows the mean resultant vector of bees rewarded on the blue disc (*n* = 18, *p* = 0.11); the green arrow shows the same for bees rewarded on the green disc (*n* = 12, *p* = 0.51). **(F)** Fixation time (in seconds; median, quartiles and outliers) of the CS+ (red boxplot) and of the CS− (gray boxplot) during the post-test. **(G)** Percentage of bees choosing first either the CS+, the CS− disc or not making a choice (NC) according to the color onto which they were rewarded. The 95% confidence interval is shown in each case. **(H)** Circular distribution of orientation vectors of learners (i.e., bees that chose first the CS+ in the post-test) during the post-test. The black arrow inside the distribution is the mean resultant vector of the entire groups of learners (*p* = 17, < 0.0001). The blue arrow shows the mean resultant vector of learners rewarded on the blue disc (*p* = 11, *n* = 0.003); the green arrow shows the same for learners rewarded on the green disc (*n* = 6, *p* = 0.002). **(I)** Fixation time (in seconds; median, quartiles and outliers) of the CS+ (red boxplot) and of the CS− (gray boxplot) by learners during the post-test. **(A,D,G)** Different lower-case letters above bars indicate significant differences (*p* < 0.05). The position of the stimuli was varied between bees during the pre-test; data were normalized to display always the CS+ on the left side and the CS− on the right side. **(C,F,I)** Bees were considered as fixating a stimulus when they were at 90° ± 20° for the CS+ and −90° ± 20° for the CS−. *p < 0.05; **p < 0.001, ***p < 0.0001; NS, non-significant.

Figure 3 shows the performance of bees in the VR setup under closed-loop conditions. In the *pre-test*, all bees (*n* = 30) made a decision: the proportion of bees spontaneously choosing the blue disc was 40% while that choosing the green disc was 60%. These proportions did not differ significantly from each other (Figure 3A; GLM binomial family; blue disc vs. green disc: z_59_ = 1.87, *p* = 0.06). Yet, when choices were analyzed in terms of a side bias, more bees oriented spontaneously towards the right than to the left side (z_59_ = −2.28, *p* = 0.02). This was overcome during training, as the side of the rewarding stimulus was randomized from bee to bee.

During *training*, bees were rewarded with sucrose solution on the stimulus they did not prefer in the pre-test (which became the CS+) and punished with quinine solution on the stimulus they preferred (which became the CS−). Due to this criterion, the distribution of orientation vectors exhibited by bees during the pre-test was biased towards the subsequent CS− (Figure 3B; V test, *p* < 0.0001) and more time was spent fixating it than the CS+ (Figure 3C; Wilcoxon *U* test, *U* = 477, *p* < 0.0001).

After the 12 conditioning trials, a *post-test* allowed determining whether bees (*n* = 30) reverted their color preference because of learning. During this post-test (Figure 3D), the proportions of bees choosing first the CS+, the CS− or not choosing any stimulus were 56.6%, 30% and 13.4%, respectively. The former was significantly higher than the two latter (Figure 3D; CS+ vs. CS− : z_88_ = 2.13, *p* = 0.02; CS+ vs. no choice: z_88_ = −2.99, *p* = 0.003; CS− vs. no choice: z_88_ = −0.87, *p* = 0.38). Discriminating learning success according to which color was rewarded (Figure 3G) showed that when the blue disc was the CS+, the proportions of bees choosing the CS+, the CS− or not choosing any stimulus were 61.1%, 16.7% and 22.2%, respectively. The former was significantly higher than the other two (CS+ vs. CS− : z_53_ = 2.59, *p* = 0.01; CS+ vs. no choice: z_53_ = −2.28, *p* = 0.02; CS− vs. no choice: z_53_ = 0.42, *p* = 0.67). When the green disc was the CS+, the proportions of bees choosing the CS+, the CS− or not choosing any stimulus were 50%, 41.7% and 8.3%, respectively. In this case, the CS+ proportion was not different from the CS− one but differed from the no-choice proportion (CS+ vs. CS−: z_35_ = 0.41, *p* = 0.68; CS+ vs. no choice: z_35_ = −2.01, *p* = 0.04; CS− vs. no choice: z_35_ = −1.72, *p* = 0.08). Thus, learning was more effective for the blue color than for the green color. In other words, reverting the pre-test color preference from green to blue was more effective than from blue to green.

The distribution of orientation vectors during the post-test was not significantly directed towards the CS + (Figure 3E, Rayleigh test, *p* = 0.18) irrespective of the nature of the positively reinforced stimulus (blue rewarded: *p* = 0.11, green rewarded: *p* = 0.51). Furthermore, bees did not spend more time fixating the CS+ (Figure 3F; *U* = 131, *p* = 0.26). Both results seem to contradict a learning effect; yet, the mean direction of trained bees changed significantly between pre- and post-test as it was no longer oriented towards the CS− (Figures 3B,E; Watson-Wheeler test, *F* = 18.02, *p* < 0.0001), thus revealing a training-induced change also at this level.

As these post-test analyses included performances from learners (i.e., bees that chose the CS+ in the post-test) and non-learners (i.e., bees that chose the CS− in the post-test), and this could have obscured the significance of the learning effects resulting from the training, we focused on the post-test performances of learners (*n* = 17). Learners were significantly oriented toward the CS+ (Figure 3H, Rayleigh test, *p* < 0.0001) irrespective of the nature of the positively reinforced stimulus (blue rewarded: *p* = 0.003, green rewarded: *p* = 0.002), and spent significantly more time fixating the CS+ (Figure 3I; U = 510, *p* < 0.0001).

These results confirm that under closed-loop conditions, a significant proportion of bees learned to revert their original color preferences and oriented towards the CS+, which they fixated longer during the post-test.

#### Transfer Test in the Y-Maze

**Figure 4:**
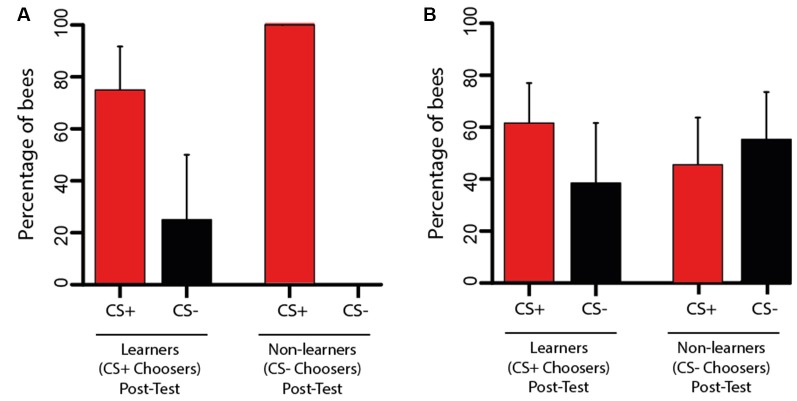
Performanceduring the transfer test of Experiments 1 and 2. **(A)** Transfer test from Experiment 1. Bees trained in the VR setup were tested in the Y-maze. Bees having chosen the CS+ (learners; *n* = 12) or the CS− (non-learners; *n* = 9) in the post-test were considered for the analysis. The bars represent their choice of the CS+ or the CS− during the transfer test in terms of the percentage of bees within each choice category. The change in performance from the post-test to the transfer test was highly significant (*p* < 0.01) as more bees chose the CS+ in the transfer test. **(B)** Transfer test from Experiment 2. Bees trained in the Y-maze were tested in the VR setup. Bees having chosen the CS+ (learners; *n* = 13) or the CS− (non-learners; *n* = 11) in the post-test were considered for the analysis. The bars represent their choice of the CS+ or the CS− during the transfer test in terms of the percentage of bees within each choice category. There was no significant change of performance between the post-test and the transfer test as both the percentage of bees choosing the CS+ and that of bees choosing the CS− were halved. The 95% confidence interval is shown in each case.

Following two refreshment trials in the VR setup (see Figure 2A), bees experienced a *transfer test* inside the Y-maze where they recovered complete freedom of movement. They had to choose between the color discs previously trained, now projected on the back walls of the maze (Figure 2A). We focused our analyses on learners (*n* = 17) and on non-learners (bees that chose the CS− in the post-test; *n* = 9) to determine how the transfer test affected their performance. From the 17 learners, five were excluded from the analysis as they did not make any choice during the transfer test. Figure 4A shows that from the remaining bees, 75% (*n* = 9) chose again the CS+ and 25% (*n* = 3) chose the CS− erroneously. Interestingly, 100% of the non-learners (*n* = 9) reverted their incorrect choice and chose the CS+ in the transfer test. A McNemar test including both learners and non-learners showed that the change in performance was significant (*χ*^2^ = 7.11, df: 1, *p* < 0.01).

Thus, performance was improved following the change of context between VR and Y-maze, in particular because the transfer revealed that the bees originally labeled as non-learners had learned the visual discrimination. The two refreshment trials performed after the post-test cannot account for this change as we observed in parallel a 25% reduction in the proportion of learners following these refreshment trials. We conclude that granting bees with the possibility of free movement and enhanced active vision improved the expression of visual learning.

### Experiment 2: From the Y-maze to VR

#### Performance in the Y-Maze

**Figure 5:**
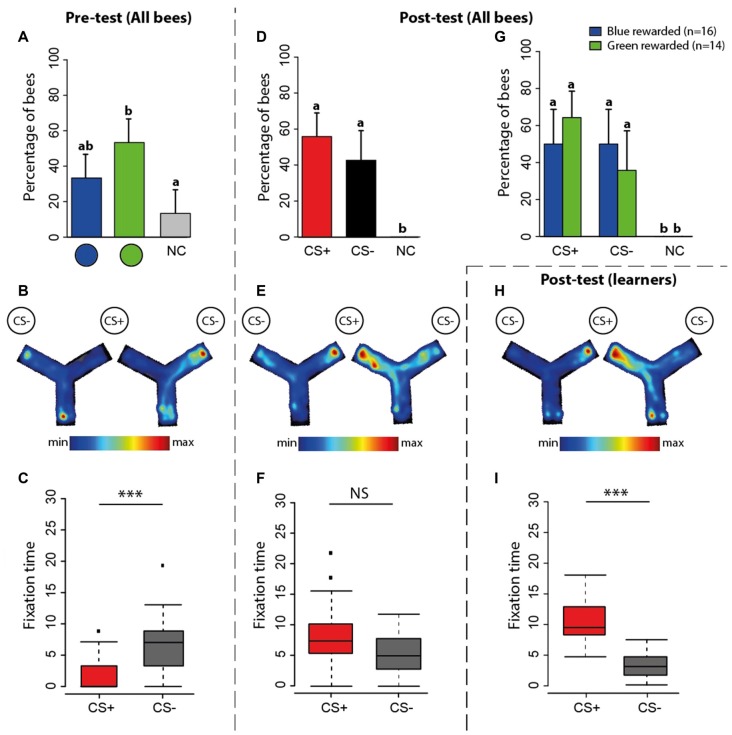
Experiment 2: spontaneous preferences (pre-test) and learning-induced preferences (post-test) at the Y-maze. **(A)** Percentage of bees (*n* = 30) either choosing first the green disc, the blue disc or not making a choice (NC) during the pre-test in the maze. **(B)** Pooled heatmap representing the normalized mean time spent within the maze during the pre-test. Although at this stage of the experiment there is neither a CS+ nor a CS−, we use these terms to indicate the stimuli that will become CS+ and CS− during the subsequent training. The stimulus that was more attractive to bees became the CS− and the less attractive became the CS+. **(C)** Time spent (in seconds; median, quartiles and outliers) in the arm of the CS+ (red boxplot) and of the CS− (gray boxplot) during the pre-test. **(D)** Percentage of bees (*n* = 30) choosing first either the CS+, the CS− disc or not making a choice (NC) during the post-test. The 95% confidence interval is shown in each case. **(E)** Pooled heatmap representing the normalized mean time spent within the maze during the post-test. The position of the CS+ and CS− is indicated. **(F)** Time spent (in seconds; median, quartiles and outliers) in the arm of the CS+ (red boxplot) and of the CS− (gray boxplot) during the post-test. **(G)** Percentage of bees choosing first either the CS+, the CS− disc or not making a choice (NC) according to the color onto which they were rewarded. The 95% confidence interval is shown in each case. **(H)** Pooled heatmap representing the normalized mean time spent by learners within the maze during the post-test (*n* = 17). **(I)** Time spent (in seconds; median, quartiles and outliers) in the arm of the CS+ (red boxplot) and of the CS− (gray boxplot) by learners (*n* = 17) during the post-test. **(A,D,G)** Different lower-case letters above bars indicate significant differences (*p* < 0.05). **(B,E,H)** The position of the CS+ and the CS− was varied during training; the left maze corresponds to a trial in which the CS+ was presented on the right and the CS− on the left; the right maze shows the reversed situation. **(C,F,I)** *p < 0.05; **p < 0.001, ***p < 0.0001; NS, non-significant.

Figure 5 shows the performance of bees in the Y-maze. During the *pre-test* (Figure 5A), 33.3% and 53.3% of the bees preferred the blue disc and the green disc, respectively; 13.3% of the bees did not choose any stimulus. Despite the apparent preference for the green disc, no difference was found between the percentages of bees choosing the blue vs. the green disc (Figure 5A; blue vs. green: z_89_ = 1.55, *p* = 0.12; blue vs. no choice: z_89_ = −1.78, *p* = 0.07; green vs. no choice: z_89_ = −3.08, *p* = 0.002). An analysis of trajectories within the Y-maze allowed establishing a heat map representing the normalized mean time spent within the maze during the pre-test (Figure 5B). The pooled heat map showed that bees spent more time in the arm displaying the color that was subsequently designated as CS− during training. Bees also spent significantly more time fixating this stimulus during the pre-test (Figure 5C; U = 29, *p* = 0.0001).

During *training*, bees were rewarded with sucrose solution on the stimulus (CS+) they did no prefer in the pre-test and punished with quinine solution on the stimulus (CS−) they preferred. After the 12 conditioning trials, a *post-test* allowed determining whether bees reverted their color preference because of learning. In this post-test, no bee remained undecided. The proportion of bees choosing first the CS+ was 57% while that of bees choosing the CS− was 43% (Figure 5D; CS+ vs. CS−: z_59_ = 1.03, *p* = 0.3). Note that the percentage of bees choosing the CS+ was similar to that observed in the post-test in the VR setup (56.6%; see Figure 3D). A main difference between that post-test and the one in the Y-maze resides in the absence of bees not making any decision in the maze. The maze seems to have increased the percentage of bees choosing the CS−. This pattern of results was common to bees rewarded on green and on blue colors (Figure 5G). We also analyzed the time spent in the maze arms. The pooled heat map showed that during the post-test bees tended to spend more time in the CS+ arm (Figure 5E), thus confirming that despite the high percentage of bees labeled as “CS− choosers” (43%, see above), learning occurred in the Y-maze. A comparison of the time spent in the CS+ and in the CS− arms was marginally non-significant (Figure 5F; U = 306, *p* = 0.06).

To confirm that learning indeed occurred in the maze, at least for the bees having chosen the CS+ as their first choice in the post-test, we focused on the performance of these bees (“learners”, *n* = 17). Their pooled heat map indicated that they preferred to stay in the CS+ arm (Figure 5H) and the quantitative analysis of the time spent in each arm of the maze revealed a highly significant preference for the CS+ arm (Figure 5I; U = 148, *p* < 0.001). Thus, 57% of the bees did indeed learn to choose the rewarded color in the Y-maze.

#### Transfer Test in the VR Setup

After two refreshment trials in the Y-maze, bees were fixed to the tether and placed, one by one, in the VR setup for a *transfer test*. They could see in front of them the two-colored discs that were trained in the Y-maze with the difference that the degrees of freedom were reduced by the tether. We focused on learners (*n* = 17) and on non-learners (*n* = 13) to determine how the transfer test affected their performance. From the 17 learners, four were excluded from the analysis as they did not make any choice during the transfer test. From the 13 non-learners, two were excluded for the same reason. Figure 4B shows that from the remaining learners 61.54% (*n* = 8) chose again the CS+ and 38.46% (*n* = 5) chose the CS− erroneously. In the case of the non-learners, 45.45% (*n* = 5) reverted their choice and chose the CS+ while 54.54% (*n* = 6) persisted in choosing the CS− incorrectly. A McNemar test including both learners and non-learners showed that there was no significant change in performance between the post-test and the transfer test (*χ*^2^ = 0.07, df: 1, *p* = 0.79).

Thus, the transfer from the Y-maze to the VR setup induced unpredictable performances in both groups of bees; i.e., a learner could become a non-learner with practically the same probability of remaining a learner, and the same occurred with a non-learner. This shows that constraining movements and impairing active vision influence the expression of visual learning.

### Comparison of Acquisition Performances in Experiments 1 and 2

The results of both transfer experiments indicate that the expression of learning was improved (Experiment 1) or impaired (Experiment 2) depending on the direction of transfer but that acquisition success was similar, at least when the % of bees choosing the CS+ in the post-test of both experiments was considered. Yet, to conclude that this was the case, an analysis of the dynamics of acquisition during the learning trials is necessary.

**Figure 6:**
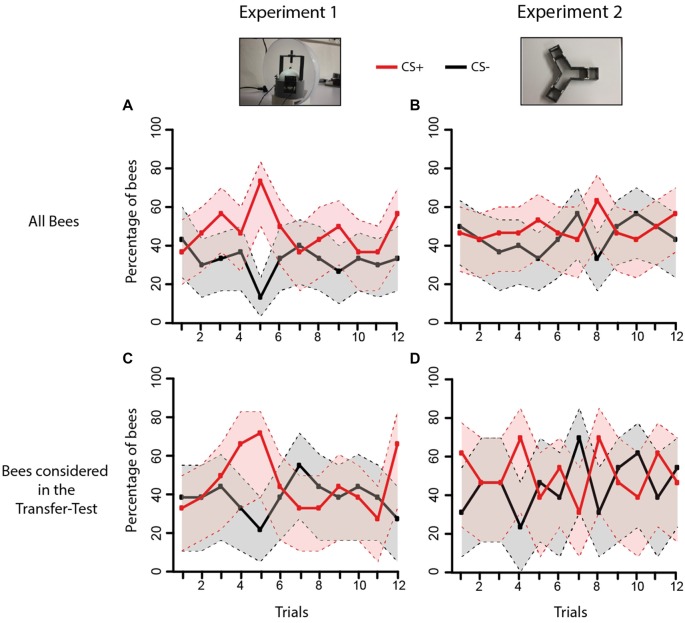
Acquisition performance of bees during Experiments 1 and 2. The graphs show the proportion of bees choosing first the CS+ (red curve) or the CS− (black curve) during the 12 conditioning trials. **(A)** Acquisition performance of all bees trained in Experiment 1 in the VR setu*p* (*n* = 30); **(B)** acquisition performance of all bees trained in Experiment 2 in the Y-maze (*n* = 30). **(C)** Same as in **(A)** but only for bees that made a choice in the post-test and whose performances were consequently analyzed in the transfer test (*n* = 21; see Figure 4A). **(D)** Same as in **(B)** but only for bees that made a choice in the post-test and whose performances were analyzed in the transfer test (*n* = 24; see Figure 4B). The gray and pink areas around the curves represent the 95% confidence interval of CS+ and CS− choices, respectively.

In both experiments, bees experienced 12 successive conditioning trials. Figures 6A,B show the acquisition performances of all bees trained in Experiments 1 and 2, respectively. Supplementary Tables S2, S3 show the individual performances of the bees in terms of their responses to the CS+, the CS− and the absence of choice along trials of Experiments 1 and 2, respectively. Performances were not significantly different between experiments (Experiment effect: *χ*^2^ = 0.07, df: 1, *p* = 0.79). There were neither differences according to the CS (CS effect: *χ*^2^ = 0.07, df: 1, *p* = 0.79) nor to trial (Trial effect: *χ*^2^ = 4.80, df: 11, *p* = 0.94). Accordingly, the global dynamics of both experiments did not differ (Interaction Experiment*CS*Trial: *χ*^2^ = 45.37, df: 34, *p* = 0.09). Interestingly, the learning curves of both experiments show that discrimination was apparently reached in trial 5, but that with further trials it was no longer visible, even if the post-tests showed that a significant percentage of bees learned the discrimination. Focusing on individual performances (Supplementary Tables S2, S3) did not allow detecting particular strategies followed by bees. For instance, bees categorized as learners for their correct choice of the CS+ in the post-test did not necessarily perform correctly in the last acquisition trial.

Restraining the analysis to the bees whose performances were analyzed in the transfer tests (i.e., bees that chose either the CS+ or the CS− in the respective post-tests) showed again that learning did not differ between experiments (Figures 6C,D: Experiment effect: *χ*^2^ = 0.52, df: 1, *p* = 0.47). There were neither significant difference according to the CS effect (*χ*^2^ = 0.07, df: 1, *p* = 0.79) nor to the trial effect (*χ*^2^ = 9.44, df: 11, *p* = 0.58). The global dynamics of both experiments was, therefore, not different (Interaction Experiment*CS*Trial: *χ*^2^ = 40.15, df: 34, *p* = 0.22). A similar result was obtained if the analysis was restrained only to learners (Supplementary Figure S2A) or to non-learners (Supplementary Figure S2B). In the case of learners, no difference in acquisition was found between experiments (Experiment effect: *χ*^2^ = 0.05, df: 1, *p* = 0.82) but again, discrimination in trial 5 was maximal. There were neither differences according to the CS (CS effect: *χ*^2^ = 0, df:1, *p* = 1) nor to trial (Trial effect: *χ*^2^ = 6.03, df: 11, *n* = 0.87). The global dynamics of both experiments did not differ in the case of learners (Experiment*CS*Trial: *χ*^2^ = 29.16, df: 34, *p* = 0.7). In the case of non-learners (Supplementary Figures S2C,D, acquisition did also not differ between experiments (Experiment effect: *χ*^2^ = 0.49, df: 1, *p* = 0.49) and there were neither differences according to the CS (CS effect: *χ*^2^ = 0.39, df: 1, *p* = 0.53) nor to trial (Trial effect: *χ*^2^ = 4.61, df: 11, *p* = 0.95). For non-learners, the global dynamics of both experiments did not differ (Interaction Experiment*CS*Trial: *χ*^2^ = 30.14, df: 34, *p* = 0.66).

These results show that despite the differences in movement freedom and access to active vision between the different contexts of Experiments 1 and 2, performances during training were similar. The acquisition curves did not provide clear evidence of learning, even if in both experiments 57% of bees learned the discrimination, as revealed by the post-tests following training. In any case, the fact that transfer performances differed depending on transfer direction (Figure 4) indicates that bees may have learned the visual discrimination differently, as revealed by the different sensitivity to a change in context.

## Discussion

We found that acquisition of a simple color discrimination was possible both in a VR and in a real environment. Even if learning curves did not provide clear evidence of discrimination learning, bees learned the task with a similar success in both contexts. There were more bees choosing the CS− in the post-test of Experiment 2 but this increase was at the expense of bees not making any decision in the post-test of Experiment 1; otherwise, the percentage of bees choosing the CS+ (57%) was the same in both experiments (compare Figures 3D, 5D). Yet, the transfer of information learned between contexts was asymmetric: bees trained in the VR setup improved their performances when moved to the Y-maze, while transfer from the Y-maze to the VR setup induced inconsistent performances. These results reveal that despite apparent similarities in acquisition, bees may have learned different visual cues in VR and in the Y-maze. They also underline the importance of free movements and active vision while performing a visual discrimination.

Active vision is the capacity to vary the observer’s viewpoint to scan the environment and extract better information from it. Motor processes are necessary for achieving this task (Findlay and Gilchrist, 2003) and they are therefore important for visual identification and location of objects in a scene. Motion detection is necessary to interpret the spatiotemporal flow of information that arises when an animal moves within a complex environment and that is detected by its visual system (Borst and Egelhaaf, 1989; Srinivasan et al., 1999; Egelhaaf et al., 2012). Flying insects face the challenge of extracting adaptive information from this continuous visual input occurring at the high speed imposed by flight performances (Zeil et al., 1996; Egelhaaf and Kern, 2002; Egelhaaf et al., 2012). Bees, flies and other flying insects actively shape the dynamics of the image flow on their eyes, a strategy that facilitates the solving of spatial vision tasks (Zeil, 1993a,b, 1997; Zeil et al., 1996; Srinivasan, 1998; Srinivasan and Zhang, 2004). In this context, active vision is crucial to segregate rotational from translational optic flow components. In the case of object detection, motion contrast is particularly relevant at the object borders. When an object protrudes from its background, motion-parallax cues are detectable at its borders, thus differentiating it from the background in terms of retinal speed: the object seems to move faster than the background (or slower, if it is located on a plane behind that of a foreground). Many insect species, including honey bees, use this relative motion to detect objects and to infer their distance (Lehrer et al., 1988, 1990; Srinivasan et al., 1989). In bees, relative motion is processed via the L-receptor (“green” receptor) channel, which provides an achromatic pathway for motion detection (Lehrer et al., 1990). In our experiments, the colored stimuli to be learned were projected onto the screens of our setups, thus lying flat on their respective backgrounds. For such stimuli, L-receptor contrast with respect to the background is also important for edge detection and shape discrimination (Lehrer et al., 1990; Lehrer and Srinivasan, 1993; Hempel de Ibarra and Giurfa, 2003). Thus, to better perceive and learn our color discs, bees need to detect their contrasting borders via their L-receptor contrast. Our stimuli provided such a contrast relative to the background, the semi-transparent screen onto which they were projected (blue disc: 0.33; green disc: 0.90; see Supplementary Table S1), for a bee to scan the stimuli and their edges.

The task to which the bees were trained could therefore rely on two main cues. One is the chromatic contrast (color difference) between the green and the blue disc. These two colors can be easily differentiated by bees as they occupy different distant loci in the bee color space (14.7 COC units; Menzel and Backhaus, 1991). Bees could thus learn the chromatic difference by focusing on the colored area of the discs. The other cue is the L-receptor contrast, which has been shown to contribute to color-stimulus discrimination and detectability (Giurfa et al., 1996, 1997). As mentioned above (see also Supplementary Table S1), it facilitates edge detection and thus a better perception of the stimulus global shape (Hempel de Ibarra and Giurfa, 2003). Typically, bees scan edges actively in order to apprehend stimulus shape (Lehrer et al., 1990). Total stimulus intensity also differed between the blue and the green disc (3363 μWatt/cm^2^ and 2950 μWatt/cm^2^, respectively); yet, numerous works have shown that total intensity is not taken into account by bees during color discrimination tasks (Backhaus, 1991; Chittka et al., 1992; Brandt and Vorobyev, 1997).

In the Y-maze, where bees could freely move and actively scan stimulus edges, discrimination learning could have thus relied on both the chromatic and the L-receptor contrast, which together could contribute to efficient stimulus differentiation. In the VR setup, both cues were in principle available, but bees were limited in their capacity to scan edges due to the tethering situation and the fact that it creates a higher resistance against movements (Catton et al., 2007). The closed-loop situation allowed updating of stimulus position but it may not have induced the same flow of visual information as the one derived from free active scanning. Thus, learning in the VR setup may have relied essentially on chromatic contrast and to a minor extent on achromatic contrast. On the contrary, in the Y-maze, bees may have used actively both cues and to a similar and complementary extent. In both scenarios, learning the discrimination was possible making use of the cues that were available. Yet, transferring them from the Y-maze to the VR setup may have implied a loss or a decrease of L-receptor contrast information, thus resulting in inconsistent performances, depending on the extent to which bees could retrieve both cues in the VR setup. By opposition, the reverse transfer may have implied a gain of this information, thus resulting in an improvement of performance in the Y-maze. This hypothesis could account for the asymmetric transfer of learning observed in our experiments. Experiments testing transfer of visual learning between the VR setup and free-flight conditions could help to gain a clearer understanding of the strategies used by bees in these different scenarios. Yet, achieving a proper transfer between free-flight and tethering conditions is difficult because when a tethered bee is moved to free-flight conditions, it may simply not return to the experimental place if the tethering is considered as a negative experience.

The acquisition curves of both experiments did not provide clear evidence of discrimination learning (Figure 6) even if the post-tests showed that at least 57% of the bees learned the discrimination (in Experiment 1 it may have been even more given the transfer performance of bees having chosen the CS− in the post-test). It thus seems that bees learned the difference between CS+ and CS− but their performance in the two setups did not reflect such learning. In other words, this deficit was common to both setups and it cannot be ascribed to particular constraints imposed by one of them. A possible explanation for this result could be the amount of reward delivered along the training procedure. As 4 μl of sucrose solution were delivered per rewarding trial (see “Materials and Methods” section) and bees experienced 12 CS+ trials in both setups, they would reach the end of training with an almost filled crop (48 μl for 50–60 μl of crop capacity; Núñez, 1966). This could result in a progressive loss of appetitive motivation and thus in a loss of conditioned responses. The fact that bees reached a high stimulus differentiation on trial 5 but showed afterwards less or no evidence of discrimination agrees with this hypothesis. In the post-test, this effect could have been overcome by the absence of reward following stimulus choice. Diminishing the amount of reward provided per trial, or the number of trials, could help solving this problem.

The limited visual information available in the dark environment in which the experiments were performed cannot fully account for the fact that the level of learning reached at the end of training (57% in both experiments) was lower than that typically obtained in experiments with free-flying bees trained and tested in daylight conditions (80%–100%) (Giurfa et al., 1996, 1997). In a previous work, we characterized learning of free-flying bees trained to discriminate a green disc from a blue square in a maze similar to the one used in this work and set under similar illumination conditions (Buatois et al., 2017). In this case, bees flying freely between the hive and the maze showed 100% discrimination learning in the post-test and learning curves that were clearly segregated for the CS+ and the CS−. A first difference with the present work resides in the possibility to return to the hive to deliver the food gathered after each conditioning trial, which was not granted in our present work. In our case, bees did not return to the hive after collecting the food in each trial, thus filling progressively more their crop. As mentioned above, this could induce a loss of appetitive motivation. Note that in another conditioning protocol widely used in honey bees, the olfactory conditioning of the PER (Bitterman et al., 1983; Giurfa and Sandoz, 2012), harnessed bees learn efficiently to associate an odorant with sucrose reward without having the possibility to return to the hive between trials. Thus, the loss of social contact is probably not the main factor affecting the bees’ performance during training. Instead, the loss of appetitive motivation may be more important for the manifestation of learning.

Does tethering just affect the possibility of active vision or may it additionally induce undesirable levels of stress in the bees trained and tested in the VR setup, responsible for deficits in performances? Under VR conditions, 56.6% of the bees learned the discrimination, i.e., chose the CS+ in the post test. Notably, 100% of those that chose the CS− in the same conditions, chose the CS+ when granted with movement freedom. Thus, tethering may affect the expression of learning but not learning in itself. Furthermore, in the olfactory conditioning of the PER (Bitterman et al., 1983; Giurfa and Sandoz, 2012), which does not require the use of active vision, total immobilization does not affect at all the capacity to learn odor-sucrose associations. In a visual variant of this protocol in which visual stimuli paired with sucrose solution are used to condition the PER of harnessed bees, learning is facilitated when movement is added to the visual stimulation (Balamurali et al., 2015). Thus, when active vision is available it may help overcome the potential stress of the harnessing situation. If bees are motivated enough to obtain food, which may be controlled by prolonging the starvation period before training and testing and by delivering minute rewards during training (Matsumoto et al., 2012), they will learn appetitive associations like the ones conditioned in the VR setup.

Several aspects of the VR setup may be improved to overcome some of the limitations mentioned above. For instance, the updating of the visual panorama following the bee’s decisions could be made more realistic. Indeed, only rotational stimulus movement was allowed but no stimulus looming/receding was provided, thus suppressing a translational component that may be essential for learning in bees. In the absence of a depth dimension for stimulus variation, efferent copies generated by the bee’s motor decisions on the treadmill were only collated incompletely with the reafferent sensory input that resulted from its movements (von Holst and Mittelstaedt, 1950; von Holst, 1954; Webb, 2004). Therefore, the bee would never attain a desired goal, thus making efferent copies useless to predict the effect of its actions. This situation could create a situation akin to “frustration” or “learned helplessness”, conspiring against learning success (Yang et al., 2013; de Brito Sanchez et al., 2015; Dinges et al., 2017). We are consequently improving our setup to include looming/receding cues in correspondence with the bee’s forward or backward movements.

Training and testing tethered bees with visual discriminations in a VR environment should solve the problem of coupling behavioral analyses with invasive recordings of neural activity in the bee brain (Schultheiss et al., 2017). This was not possible until now because visual behavior was only accessible in free flying bees. Although several attempts have been done to develop visual PER conditioning (Avarguès-Weber and Mota, 2016), our results provide a more realistic and at the same time controlled scenario for studying such learning at multiple levels, from a behavioral to a molecular level. Thus, at least for the simple task trained in our work, the goal of accessing visual-neuropile activity, for instance, via electrophysiological procedures, is realistic. Multielectrodes that record local-field potentials could be implanted in visual areas such as the medulla, the lobula or the central complex, or in central integration regions such as the mushroom bodies to characterize the neural signature of this task. Future challenges should focus also on developing higher-order learning protocols to reproduce in a controlled VR environment the cognitive feats of bees which have firmly established their reputation as a model for cognition.

## Author Contributions

AB, AA-W and MG designed the experiments. The experiments were conducted by AB and CF. AB analyzed the results and prepared figures and tables. AB, AA-W, PS and MG wrote the manuscript. All authors reviewed and approved the final manuscript.

## Conflict of Interest Statement

The authors declare that the research was conducted in the absence of any commercial or financial relationships that could be construed as a potential conflict of interest. The reviewer MD declared a past supervisory role with one of the authors AB to the handling Editor, who ensured that the process met the standards of a fair and objective review.

## Footnotes

^1^https://www.r-project.org/

## References

[B2] Avarguès-Weber A. Deisig N. Giurfa M. (2011). Visual cognition in social insects. Annu. Rev. Entomol. 56, 423–443. 10.1146/annurev-ento-120709-144855 20868283

[B3] Avarguès-Weber A. Dyer A. G. Combe M. Giurfa M. (2012). Simultaneous mastering of two abstract concepts by the miniature brain of bees. Proc. Natl. Acad. Sci. U S A 109, 7481–7486. 10.1073/pnas.1202576109 22517740 PMC3358847

[B1] Avarguès-Weber A. Mota T. (2016). Advances and limitations of visual conditioning protocols in harnessed bees. J. Physiol. Paris 110, 107–118. 10.1016/j.jphysparis.2016.12.006 27998810

[B4] Avarguès-Weber A. Portelli G. Benard J. Dyer A. Giurfa M. (2010). Configural processing enables discrimination and categorization of face-like stimuli in honeybees. J. Exp. Biol. 213, 593–601. 10.1242/jeb.039263 20118310

[B5] Backhaus W. (1991). Color opponent coding in the visual system of the honeybee. Vision Res. 31, 1381–1397. 10.1016/0042-6989(91)90059-e 1891826

[B6] Balamurali G. S. Somanathan H. Hempel de Ibarra N. (2015). Motion cues improve the performance of harnessed bees in a colour learning task. J. Comp. Physiol. A 201, 505–511. 10.1007/s00359-015-0994-7 25739517

[B7] Benard J. Stach S. Giurfa M. (2006). Categorization of visual stimuli in the honeybee *Apis mellifera*. Anim. Cogn. 9, 257–270. 10.1007/s10071-006-0032-9 16909238

[B8] Bitterman M. E. Menzel R. Fietz A. Schäfer S. (1983). Classical conditioning of proboscis extension in honeybees (*Apis mellifera*). J. Comp. Psychol. 97, 107–119. 10.1037//0735-7036.97.2.107 6872507

[B9] Borst A. Egelhaaf M. (1989). Principles of visual motion detection. Trends Neurosci. 12, 297–306. 10.1016/0166-2236(89)90010-6 2475948

[B10] Brandt R. Vorobyev M. (1997). Metric analysis of threshold spectral sensitivity in the honeybee. Vision Res. 37, 425–439. 10.1016/s0042-6989(96)00195-2 9156174

[B11] Brembs B. Heisenberg M. (2000). The operant and the classical in conditioned orientation of *Drosophila melanogaster* at the flight simulator. Learn. Mem. 7, 104–115. 10.1101/lm.7.2.104 10753977 PMC311324

[B12] Buatois A. Pichot M. C. Schultheiss P. Sandoz J. C. Lazzari C. Chittka L.. (2017). Associative visual learning by tethered bees in a controlled visual environment. Sci. Rep. 7:127903. 10.1038/s41598-017-12631-w 29018218 PMC5635106

[B13] Catton K. B. Webster D. R. Brown J. Yen J. (2007). Quantitative analysis of tethered and free-swimming copepodid flow fields. J. Exp. Biol. 210, 299–310. 10.1242/jeb.02633 17210966

[B14] Chittka L. (1998). Sensorimotor learning in bumblebees: long-term retention and reversal training. J. Exp. Biol. 201, 515–524.

[B15] Chittka L. Beier W. Hertel H. Steinmann E. Menzel R. (1992). Opponent colour coding is a universal strategy to evaluate the photoreceptor inputs in Hymenoptera. J. Comp. Physiol. A 170, 545–563. 10.1007/bf00199332 1507155

[B16] de Brito Sanchez M. G. Serre M. Avarguès-Weber A. Dyer A. G. Giurfa M. (2015). Learning context modulates aversive taste strength in honey bees. J. Exp. Biol. 218, 949–959. 10.1242/jeb.117333 25788729

[B17] Dinges C. W. Varnon C. A. Cota L. D. Slykerman S. Abramson C. I. (2017). Studies of learned helplessness in honey bees (*Apis mellifera ligustica*). J. Exp. Psychol. Anim. Learn. Cogn. 43, 147–158. 10.1037/xan0000133 28191986

[B18] Dobrin S. E. Fahrbach S. E. (2012). Visual associative learning in restrained honey bees with intact antennae. PLoS One 7:e37666. 10.1371/journal.pone.0037666 22701575 PMC3368934

[B19] Dyer A. G. (2012). The mysterious cognitive abilities of bees: why models of visual processing need to consider experience and individual differences in animal performance. J. Exp. Biol. 215, 387–395. 10.1242/jeb.038190 22246247

[B21] Egelhaaf M. Boeddeker N. Kern R. Kurtz R. Lindemann J. P. (2012). Spatial vision in insects is facilitated by shaping the dynamics of visual input through behavioral action. Front. Neural Circuits 6:108. 10.3389/fncir.2012.00108 23269913 PMC3526811

[B20] Egelhaaf M. Kern R. (2002). Vision in flying insects. Curr. Opin. Neurobiol. 12, 699–706. 10.1016/s0959-4388(02)00390-2 12490262

[B23] Erber J. Pribbenow B. Bauer A. Kloppenburg P. (1993). Antennal reflexes in the honeybee: tools for studying the nervous-system. Apidologie 24, 283–296. 10.1051/apido:19930308

[B22] Erber J. Schildberger K. (1980). Conditioning of an antennal reflex to visual-stimuli in bees (*Apis-mellifera* L.). J. Comp. Physiol. 135, 217–225. 10.1007/bf00657249

[B24] Findlay J. M. Gilchrist I. D. (2003). Active Vision: The Psychology of Looking and Seeing. New York, NY: Oxford University Press.

[B25] Giurfa M. (2013). Cognition with few neurons: higher-order learning in insects. Trends Neurosci. 36, 285–294. 10.1016/j.tins.2012.12.011 23375772

[B26] Giurfa M. Menzel R. (1997). Insect visual perception: complex abilities of simple nervous systems. Curr. Opin. Neurobiol. 7, 505–513. 10.1016/s0959-4388(97)80030-x 9287201

[B27] Giurfa M. Sandoz J. C. (2012). Invertebrate learning and memory: fifty years of olfactory conditioning of the proboscis extension response in honeybees. Learn. Mem. 19, 54–66. 10.1101/lm.024711.111 22251890

[B28] Giurfa M. Vorobyev M. Brandt R. Posner B. Menzel R. (1997). Discrimination of coloured stimuli by honeybees: alternative use of achromatic and chromatic signals. J. Comp. Physiol. A 180, 235–243. 10.1007/s003590050044

[B29] Giurfa M. Vorobyev M. Kevan P. Menzel R. (1996). Detection of coloured stimuli by honeybees: minimum visual angles and receptor specific contrasts. J. Comp. Physiol. A 178, 699–709. 10.1007/bf00227381

[B30] Giurfa M. Zhang S. Jenett A. Menzel R. Srinivasan M. V. (2001). The concepts of ‘sameness’ and ‘difference’ in an insect. Nature 410, 930–933. 10.1038/35073582 11309617

[B31] Götz K. G. (1964). Optomotorische Untersuchung des visuellen Systems einiger Augenmutanten der Fruchtfliege *Drosophila*. Kybernetik 2, 77–92. 10.1007/bf00288561s 5833196

[B33] Heisenberg M. Wolf R. (1988). Reafferent control of optomotor yaw torque in *Drosophila melanogaster*. J. Comp. Physiol. A 163, 373–388. 10.1007/bf00604013

[B34] Hempel de Ibarra N. Giurfa M. (2003). Discrimination of closed coloured shapes requires only contrast to the long wavelength receptor. Anim. Behav. 66, 903–910. 10.1006/anbe.2003.2269

[B35] Hertz M. (1935). Die Untersuchungen üeber den Formensinn der Honigbiene. Naturwissenschaften 36, 618–624. 10.1007/bf01493245

[B37] Hori S. Takeuchi H. Arikawa K. Kinoshita M. Ichikawa N. Sasaki M.. (2006). Associative visual learning, color discrimination, and chromatic adaptation in the harnessed honeybee *Apis mellifera* L. J. Comp. Physiol. A 192, 691–700. 10.1007/s00359-005-0091-4 16425063

[B36] Hori S. Takeuchi H. Kubo T. (2007). Associative learning and discrimination of motion cues in the harnessed honeybee *Apis mellifera* L. J. Comp. Physiol. A Neuroethol. Sens. Neural Behav. Physiol. 193, 825–833. 10.1007/s00359-007-0234-x 17534629

[B38] Lehrer M. Srinivasan M. V. (1993). Object detection by honeybees: why do they land on edges? J. Comp. Physiol. A 173, 23–32. 10.1007/bf00209615

[B39] Lehrer M. Srinivasan M. V. Zhang S. W. (1990). Visual edge detection in the honeybee and its chromatic properties. Proc. Biol. Sci. B 238, 321–330. 10.1098/rspb.1990.0002

[B40] Lehrer M. Srinivasan M. V. Zhang S. W. Horridge G. A. (1988). Motion cues provide the bee’s visual world with a third dimension. Nature 332, 356–357. 10.1038/332356a0

[B41] Liu G. Seiler H. Wen A. Zars T. Ito K. Wolf R.. (2006). Distinct memory traces for two visual features in the *Drosophila* brain. Nature 439, 551–556. 10.1038/nature04381 16452971

[B42] Liu L. Wolf R. Ernst R. Heisenberg M. (1999). Context generalization in *Drosophila* visual learning requires the mushroom bodies. Nature 400, 753–756. 10.1038/23456 10466722

[B43] Luu T. Cheung A. Ball D. Srinivasan M. V. (2011). Honeybee flight: a novel ‘streamlining’ response. J. Exp. Biol. 214, 2215–2225. 10.1242/jeb.050310 21653815

[B44] Matsumoto Y. Menzel R. Sandoz J. C. Giurfa M. (2012). Revisiting olfactory classical conditioning of the proboscis extension response in honey bees: a step towards standardized procedures. J. Neurosci. Methods 211, 159–167. 10.1016/j.jneumeth.2012.08.018 22960052

[B45] Menzel R. Backhaus W. (1991). “Colour vision in insects,” in Vision and Visual Dysfunction: The Perception of Colour, ed. Gouras P. (London: MacMillan Press), 262–288.

[B46] Mota T. Giurfa M. Sandoz J. C. (2011). Color modulates olfactory learning in honeybees by an occasion-setting mechanism. Learn. Mem. 18, 144–155. 10.1101/lm.2073511 21330377

[B47] Núñez J. A. (1966). Quantitative Beziehungen zwischen den Eigenschaften von Futterquellen und dem Verhalten von Sammelbienen. Z. Physiol. 53, 142–164.

[B48] Pan Y. Zhou Y. Guo C. Gong H. Gong Z. Liu L. (2009). Differential roles of the fan-shaped body and the ellipsoid body in *Drosophila* visual pattern memory. Learn. Mem. 16, 289–295. 10.1101/lm.1331809 19389914

[B49] Rusch C. Roth E. Vinauger C. Riffell J. A. (2017). Honeybees in a virtual reality environment learn unique combinations of colour and shape. J. Exp. Biol. 220, 3478–3487. 10.1242/jeb.173062 28751492

[B50] Schultheiss P. Buatois A. Avarguès-Weber A. Giurfa M. (2017). Using virtual reality to study visual performances of honeybees. Curr. Opin. Insect. Sci. 24, 43–50. 10.1016/j.cois.2017.08.003 29208222

[B51] Srinivasan M. V. (1998). Insects as gibsonian animals. Ecol. Psychol. 10, 251–270. 10.1207/s15326969eco103&4_5

[B52] Srinivasan M. V. (2010). Honey bees as a model for vision, perception, and cognition. Annu. Rev. Entomol. 55, 267–284. 10.1146/annurev.ento.010908.164537 19728835

[B54] Srinivasan M. V. Lehrer M. Zhang S. W. Horridge G. A. (1989). How honeybees measure their distance from objects of unknown size. J. Comp. Physiol. A 165, 605–613. 10.1007/bf00610992

[B55] Srinivasan M. V. Poteser M. Kral K. (1999). Motion detection in insect orientation and navigation. Vision Res. 39, 2749–2766. 10.1016/s0042-6989(99)00002-4 10492835

[B53] Srinivasan M. V. Zhang S. (2004). Visual motor computations in insects. Annu. Rev. Neurosci. 27, 679–696. 10.1146/annurev.neuro.27.070203.144343 15217347

[B56] Takeda K. (1961). Classical conditioned response in the honey bee. J. Insect Physiol. 6, 168–179. 10.1016/0022-1910(61)90060-9

[B57] Tang S. M. Guo A. (2001). Choice behavior of *Drosophila* facing contradictory visual cues. Science 294, 1543–1547. 10.1126/science.1058237 11711680

[B32] van Hateren J. H. Srinivasan M. V. Wait P. B. (1990). Pattern recognition in bees: orientation discrimination. J. Comp. Physiol. A 167, 649–654. 10.1007/bf00192658

[B58] von Frisch K. (1914). Der Farbensinn und Formensinn der Biene. Zool Jahrb Abt Allg Zool Physiol Tiere 37, 1–238. 10.5962/bhl.title.11736

[B59] von Holst E. (1954). Relations between the central nervous system and the peripheral organs. Br. J. Anim. Behav. 2, 89–94. 10.1016/s0950-5601(54)80044-x

[B60] von Holst E. Mittelstaedt H. (1950). “The reafference principle. Interaction between the central nervous system and the periphery,” in Selected Papers of Erich von Holst: The Behavioural Physiology of Animals and Man (London: Methuen), 139–173.

[B61] Webb B. (2004). Neural mechanisms for prediction: do insects have forward models? Trends Neurosci. 27, 278–282. 10.1016/j.tins.2004.03.004 15111010

[B62] Wolf R. Heisenberg M. (1991). Basic organization of operant-behavior as revealed in *Drosophila* flight orientation. J. Comp. Physiol. A 169, 699–705. 10.1007/bf00194898 1795235

[B63] Xi W. Peng Y. Q. Guo J. Z. Ye Y. Z. Zhang K. Yu F.. (2008). Mushroom bodies modulate salience-based selective fixation behavior in *Drosophila*. Eur. J. Neurosci. 27, 1441–1451. 10.1111/j.1460-9568.2008.06114.x 18364023

[B64] Yang Z. Bertolucci F. Wolf R. Heisenberg M. (2013). Flies cope with uncontrollable stress by learned helplessness. Curr. Biol. 23, 799–803. 10.1016/j.cub.2013.03.054 23602474

[B65] Zeil J. (1993a). Orientation flights of solitary wasps (*Cerceris*; Specidae; Hymenoptera) 1. Description of flight. J. Comp. Physiol. A 172, 189–205. 10.1007/bf00189396

[B66] Zeil J. (1993b). Orientation flights of solitary wasps (*Cerceris*; Sphecidae; Hymenoptera) 2. Similarities between orientation and return flights and the use of motion parallax. J. Comp. Physiol. A 172, 207–222. 10.1007/bf00189396

[B67] Zeil J. (1997). The control of optic flow during learning flights. J. Comp. Physiol. A 180, 25–37. 10.1007/s003590050024

[B68] Zeil J. Kelber A. Voss R. (1996). Structure and function of learning flights in bees and wasps. J. Exp. Biol. 199, 245–252. 9317729 10.1242/jeb.199.1.245

[B69] Zhang S. W. Lehrer M. Srinivasan M. V. (1998). Eye-specific learning of routes and “signposts” by walking honeybees. J. Comp. Physiol. 182, 747–754. 10.1007/s003590050219

