# Supplementary material for: Transfer of Visual Learning Between a Virtual and a Real Environment in Honey Bees: The Role of Active Vision
Source: Front Behav Neurosci. 2018 Jul 13;12:139. doi: 10.3389/fnbeh.2018.00139 (PMC6053632; doi:10.3389/fnbeh.2018.00139)
Supplement: Supplementary file 1 [file Data_Sheet_1.PDF]

Table S1: Chromatic and achromatic properties of the trained stimuli.

**Color stimulus**	**Color Distance from the background** (COC units)	**Chromatic Contrast to the alternative stimulus** (COC units)	**Receptor Contrast** (absorbed quanta relative to the background)			**Intensity** (sum of absorbed quanta relative to the background)
			UV	B	G	
Background (plastic screen)	--	--	1	1	1	3
Blue	1.75	14.7 (to green)	0.02	0.64	0.33	0.99
Green	7.1	14.7 (to blue)	0.002	0.10	0.90	1.002

Contrasts are evaluated with respect to the black background. The illumination light considered was the one provided by the videoprojector lamp.

Receptor-specific contrasts, i.e. the relative number of absorbed quanta q with respect to the black background, were calculated as:

$$q_i = \frac{\int_0^\infty I(\lambda)\, R(\lambda)\, S_i(\lambda)\, d\lambda}{\int_0^\infty I(\lambda)\, B(\lambda)\, S_i(\lambda)\, d\lambda}, \quad i = \text{uv, blue, green receptor}; \qquad (1)$$

with $I(\lambda)$ being the intensity distribution of the illuminating light of the videoprojector , $R(\lambda)$ the spectral curve of the stimulus considered (blue or green disc; see Fig. S1), $B(\lambda)$ the spectral curve of the black background (see Fig. S1) and $S_i(\lambda)$ the spectral sensitivity of the receptor with index i (Menzel and Backhaus, 1991).

Intensity contrast of a stimulus against the black background was calculated as the sum of the absorbed quanta in the three types of photoreceptors, relative to the background (see Eq. 1).

To quantify chromatic differences, the color opponent coding space proposed specifically for the honey bee (Backhaus, 1991) was used. In such a space, chromatic coordinates A and B of the stimuli were determined as:

$$A = \sum_{i=u,b,g} a_i \frac{q_i}{q_i+1}, \qquad B = \sum_{i=u,b,g} b_i \frac{q_i}{q_i+1} \qquad (2)$$

with a_i = {-9.86, 7.70, 2.16} and b_i = {-5.17, +20.25, -15.08}; i = uv, blue, green receptor.

The perceptual colour distance D between two stimuli S_1 and S_2 was calculated as the sum of the absolute differences in chromatic coordinates A and B (city block metric; Backhaus, 1991):

$$D(S_1,S_2) = |A_{S1} - A_{S2}| + |B_{S1} - B_{S2}| \qquad (3)$$

Chromatic contrast is defined as the perceptual color distance D of a stimulus (S) to the black background (Back.). Because the background coordinates in the color space used are (0,0) (Backhaus, 1991), chromatic contrast D is calculated as:

$$D(S,Back.) = |A_S| + |B_S|$$

References

Backhaus, W. (1991). Color opponent coding in the visual system of the honeybee. *Vision Res.* 31**,** 1381-1397.

Menzel, R., and Backhaus, W. (1991). "Colour Vision in Insects," in *Vision and Visual Dysfunction. The Perception of Colour.*, ed. P. Gouras. (London: MacMillan Press), 262-288.
