# Supplementary material for: Transfer of Visual Learning Between a Virtual and a Real Environment in Honey Bees: The Role of Active Vision
Source: Front Behav Neurosci. 2018 Jul 13;12:139. doi: 10.3389/fnbeh.2018.00139 (PMC6053632; doi:10.3389/fnbeh.2018.00139)
Supplement: Supplementary file 2 [file Data_Sheet_2.PDF]

Table S2: Individual performances during the acquisition phase of Experiment 1.

	Trial 1	Trial 2	Trial 3	Trial 4	Trial 5	Trial 6	Trial 7	Trial 8	Trial 9	Trial 10	Trial 11	Trial 12
Bee1	CS+	CS+	CS+	CS+	CS+	CS-	CS-	NC	NC	NC	CS-	CS-
Bee2	CS-	CS+	CS-	NC	CS+	CS+	NC	NC	NC	NC	CS+	CS-
Bee3	CS-	CS+	CS-	CS-	CS+	CS-	CS-	CS-	CS-	CS+	CS+	CS+
Bee4	CS-	CS-	CS+	CS+	CS-	NC	NC	CS+	CS+	CS-	NC	CS+
Bee5	CS+	NC	NC	CS+	CS+	CS+	CS+	NC	NC	CS+	NC	CS+
Bee6	CS-	CS+	NC	NC	NC	NC	NC	NC	NC	NC	NC	NC
Bee7	CS-	NC	CS+	CS+	CS-	CS+	NC	CS+	CS+	CS-	CS-	CS+
Bee8	CS+	CS+	CS+	NC	NC	CS+	CS+	CS+	CS+	CS+	CS+	CS+
Bee9	CS+	CS+	CS+	CS-	CS+	CS+	CS-	CS-	CS+	NC	CS+	NC
Bee10	CS+	CS-	CS-	CS-	CS-	CS-	CS+	NC	CS+	NC	NC	CS-
Bee11	NC	CS-	CS-	CS+	CS+	CS+	CS+	CS+	CS+	CS+	NC	CS+
Bee12	CS+	CS+	CS+	NC	CS+	CS+	NC	CS-	NC	CS+	CS+	CS-
Bee13	CS-	CS+	CS+	CS-	CS+	CS-	CS+	NC	NC	NC	CS-	CS-
Bee14	CS+	CS-	CS-	CS+	CS+	CS-	CS+	CS-	CS-	CS-	CS-	CS+
Bee15	CS-	CS-	CS+	CS+	CS+	CS+	CS-	CS-	CS-	CS-	NC	CS+
Bee16	NC	NC	CS+	CS-	CS+	CS+	CS+	CS+	CS+	CS-	CS-	CS+
Bee17	NC	CS+	CS-	CS+	CS+	CS+	NC	NC	CS+	CS-	CS-	CS+
Bee18	CS+	CS-	CS+	CS-	CS+	CS+	CS+	CS+	CS-	CS+	CS+	CS+
Bee19	CS+	CS-	CS+	CS-	CS+	CS-	CS+	CS+	CS+	CS-	CS-	CS+
Bee20	CS+	NC	NC	CS+	CS+	CS-	CS-	CS-	CS+	CS+	CS+	CS+
Bee21	NC	NC	CS-	CS+	CS+	CS+	CS+	CS+	CS+	CS-	CS+	CS+
Bee22	CS-	CS-	CS+	CS+	CS-	CS+	CS-	CS+	CS+	NC	CS-	CS+
Bee23	CS-	NC	CS+	CS+	CS+	CS+	CS-	CS-	CS-	CS+	CS+	CS+
Bee24	CS-	CS+	CS+	CS+	NC	NC	CS-	CS-	NC	CS-	CS-	CS-
Bee25	NC	NC	CS-	NC	NC	NC	NC	CS+	CS+	NC	NC	NC
Bee26	CS-	CS+	CS+	CS-	CS+	CS-	CS-	CS+	CS-	NC	NC	CS-
Bee27	CS+	CS-	CS-	CS-	CS+	CS-	CS-	CS-	CS-	CS+	NC	CS-
Bee28	CS-	CS+	CS+	CS-	CS+	CS-	CS-	CS-	CS-	CS+	CS+	NC
Bee29	NC	CS+	CS-	CS+	CS+	CS+	CS-	CS+	CS+	CS+	NC	CS-
Bee30	CS-	CS+	CS+	CS-	CS+	NC	CS+	CS+	CS+	CS-	CS+	CS-
CS+	36,67%	46,67%	56,67%	46,67%	73,33%	50,00%	36,67%	43,33%	50,00%	36,67%	36,67%	53,33%
CS-	43,33%	30,00%	33,33%	36,67%	13,33%	33,33%	40,00%	33,33%	26,67%	33,33%	30,00%	33,33%

The data correspond to the learning curves (CS+ vs CS- choices) shown in Fig. 6a. For each trial, the choice of the bee in the VR setup was recorded (see Materials and Methods for details) and categorized as CS+ choice (in green), CS- choice (in red) or no choice (NC, in grey). Each row corresponds to an individual bee, thus showing the succession of choices made by this

individual. The bottom rows show the percentages of CS+ and CS- choices obtained from these performances (see Fig. 6a). Learners (bees that chose the CS+ in the post-test of Experiment 1) are highlighted in yellow.
