# Supplementary material for: Transfer of Visual Learning Between a Virtual and a Real Environment in Honey Bees: The Role of Active Vision
Source: Front Behav Neurosci. 2018 Jul 13;12:139. doi: 10.3389/fnbeh.2018.00139 (PMC6053632; doi:10.3389/fnbeh.2018.00139)
Supplement: Supplementary file 4 [file Image_1.pdf]

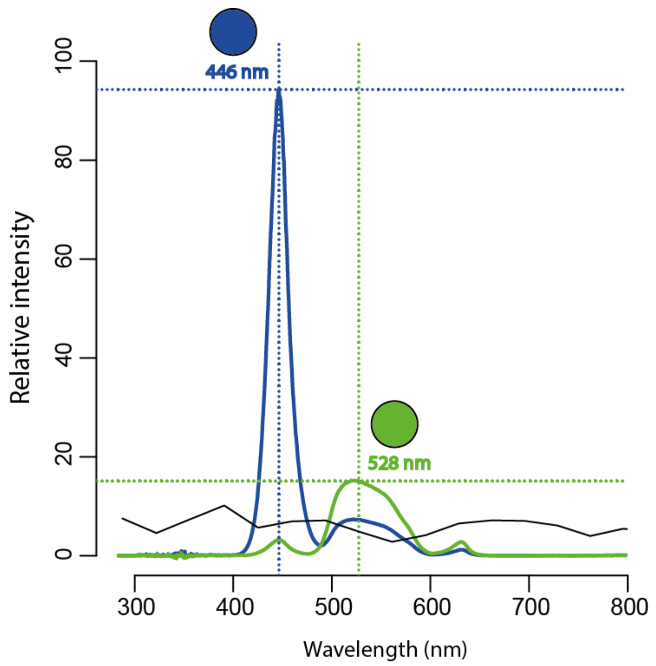

Figure S1. Spectral curves of the green (dominant wavelength 528 nm) and the blue disc (446 nm). The curve of the black background surrounding the colored discs is also shown (black curve). This curve slightly differs from a flat curve with a null intensity due to minimum amount of light produced by the videoprojector.
