# Supplementary material for: Transfer of Visual Learning Between a Virtual and a Real Environment in Honey Bees: The Role of Active Vision
Source: Front Behav Neurosci. 2018 Jul 13;12:139. doi: 10.3389/fnbeh.2018.00139 (PMC6053632; doi:10.3389/fnbeh.2018.00139)
Supplement: Supplementary file 5 [file Image_2.pdf]

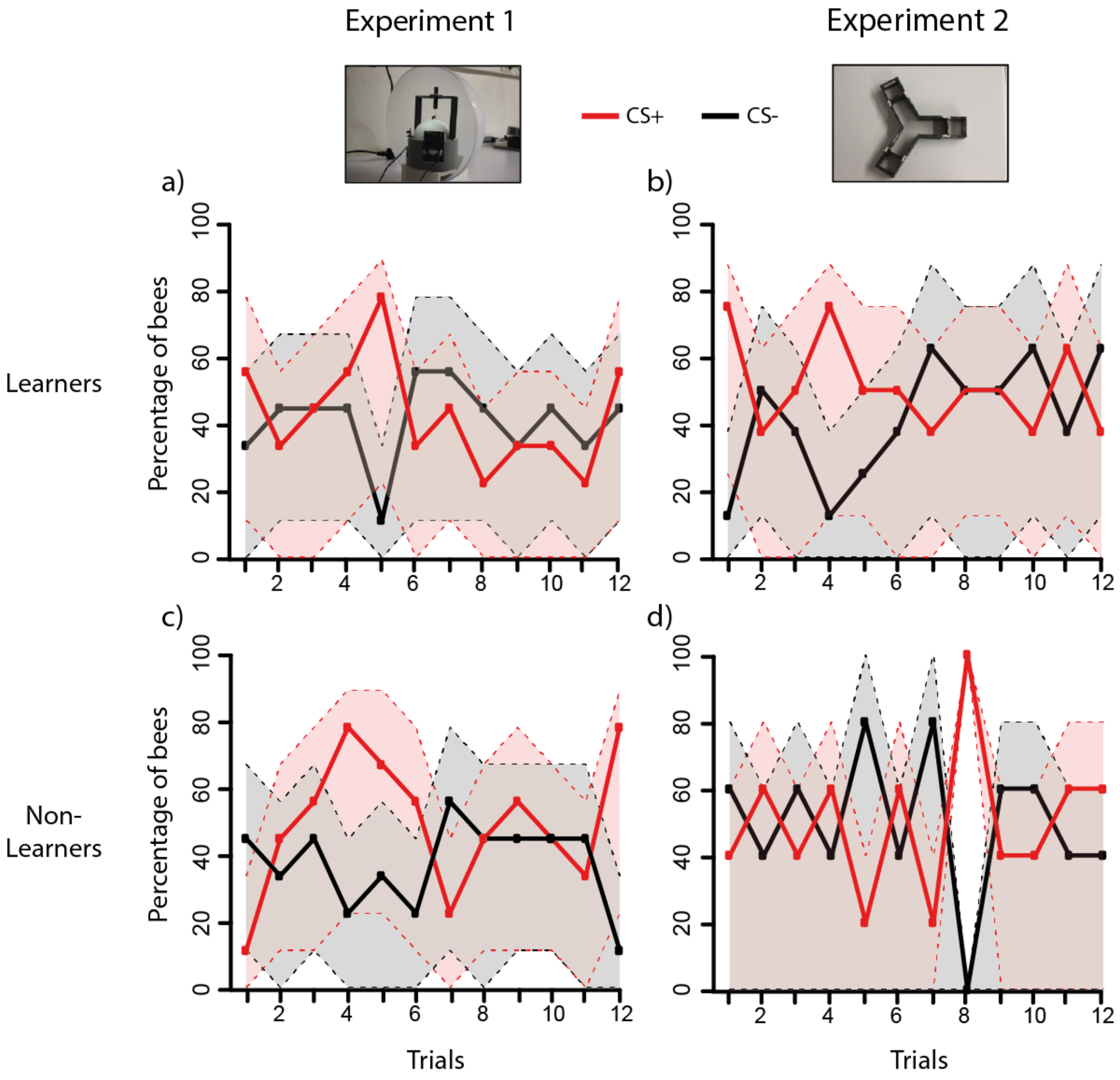

Figure S2. Acquisition performance of bees (learners and non-learners) during Experiments 1 and 2. The graphs show the proportion of bees choosing first the CS+ (red curve) or the CS- (black curve) during the twelve conditioning trials. **(a)** Acquisition performance of bees categorized as learners in the post-test of Experiment 1 in the VR setup **(**i.e. bees that chose the CS+ in the post-test of Experiment 1**)**; **(b)** acquisition performance of bees categorized as learners in the post-test of Experiment 2 in the Y-maze (i.e. bees that chose the CS+ in the post-test of Experiment 2). **(c)** Same as in **(a)** but for bees categorized as non-learners **(**i.e. bees that chose the CS- in the post-test of Experiment 1**)**. **(d)** Same as in **(b)** but for bees categorized as non-learners (i.e. bees that chose the CS- in the post-test of Experiment 2**).** Grey and pink areas around the curves represent the 95% confidence interval of CS+ and CS- choices, respectively.
